# Implementation of the Chronic Disease Care System and its association with health care costs and continuity of care in Korean adults with type 2 diabetes mellitus

**DOI:** 10.1186/s12913-018-3806-2

**Published:** 2018-12-22

**Authors:** Woorim Kim, Yoon Soo Choy, Sang Ah Lee, Eun-Cheol Park

**Affiliations:** 1 Department of Public Health, Graduate School, Yonsei University, Seoul, Republic of Korea; 2 Institute of Health Services Research, Yonsei University, Seoul, Republic of Korea; 3 Department of Preventive Medicine, Yonsei University College of Medicine, 50 Yonsei-ro, Seodaemun-gu, Seoul, 120-752 Republic of Korea

**Keywords:** Chronic disease care system, Primary health care, Health care costs, Continuity of care, Treatment continuity, Chronic disease

## Abstract

**Background:**

The Chronic Disease Care System (CDCS) has been implemented in Korea to encourage treatment continuity in chronic disease patients. This study investigated the effect of the introduction of the CDCS on health care costs and continuity of care in individuals with type 2 diabetes mellitus (T2DM).

**Methods:**

The National Health Insurance data from August, 2010 to March, 2012 (pre-policy) and from May, 2012 to December, 2013 (post-policy) were used. Introduction of the CDCS was defined as the intervention. The intervention group consisted of T2DM patients participating in the program and the control group patients not participating in the program. The Difference-in-Differences (DID) method was used to estimate the differences in total health care costs for outpatient services and continuity of care between the intervention and the control group before and after the intervention period.

**Results:**

Implementation of the CDCS was associated with decreased health care costs (β = − 46,877 Korean Won, *P* < 0.0001) and improved continuity of care (β = 0.0536, *P* < 0.0001) in the intervention group with adjustment for covariates.

**Conclusion:**

Findings confirm an association between the adoption of the CDCS and reduced health care costs and improved continuity of care. The results reveal the potential benefits of reinforcing effective chronic disease management strategies in reducing health care costs and improving treatment continuity.

## Background

Diabetes mellitus is a global public health problem, with the number of affected individuals expected to rise from 360 million in 2011 to 550 million in 2030 [1]. The prevalence of diabetes has also increased from around nine to 11% between 2011 and 2013 in South Korea, which is alarming because diabetes and its related complications often increase the burden individuals and health care systems experience in terms of health outcomes and costs [2, 3]. In fact, previous studies have reported that diabetes act to decrease the health-related quality of life (HRQoL) of affected individuals [4]. Furthermore, diabetes and its related complications are a leading cause of morbidity and mortality in Korea [5].

Specifically, the prevalence type 2 diabetes mellitus (T2DM) has been increasing noticeably at a rate of 7.9% among Korean adults [6, 7]. As diabetes is chronic in nature and can lead to severe complications, including cardiovascular disease (CVD), retinopathy, nephropathy, neuropathy, peripheral-vascular disease, and cerebrovascular disease, the quality of diabetes treatment is an important issue to address [8]. At the same time, diabetes is also known to cause around 250 million United States dollars (USD) aggregate annual direct costs [9, 10]. Moreover, patients who develop diabetes related complications are known to require higher levels of medical expenditure [9].

Considering the importance of providing proper disease management to enhance treatment quality and contain costs, the government of Korea introduced the Chronic Disease Care System (CDCS) program in 2012 [11]. The CDCS program aims to enhance care coordination by encouraging patients with T2DM or hypertension to designate a primary care clinic of their choice for continued care [12]. By voluntarily participating in the CDCS program, T2DM patients choose a preferred primary clinic and agree to consistently receive care from the selected institution. Participation assures a reduction of out-of-pocket costs for outpatient services from the regular 30 to 20% of total costs, along with the provision of health support services, such as consultation and education. Health care providers are separately provided with incentives after assessment by the Health Insurance Review and Assessment Service (HIRA).

The government expects that the CDCS program will improve treatment quality by improving continuity of care, in addition to equipping patients with better disease self-management skills. Implementation of the CDCS program has been expected to enhance continuity of care, which is significant because Korea lacks a personalized general practitioner (GP) based primary care system [13]. Additionally, because continuity of care has been associated with indicators of patient prognoses, including the development of complications, mortality, emergency room visits, and hospital admissions, promoting care coordination is also essential in maintaining health care costs [14]. Therefore, the purpose of this study was to estimate the effect of the CDCS implementation on health care costs and continuity of care in patients with T2DM.

## Methods

### Study population

This study used the Korea National Health Insurance (NHI) cohort data. In Korea, all individuals are covered by either the NHI or Medical Aid and the NHI is known to cover around 98% of the total population. The NHI cohort data were constructed based on 1,025,340 nationally representative random samples of the entire 2002 population. The sample population equals around 2.2% of the 46,605,433 residents recorded by the Korean National Health Insurance Service (KNHIS). Data were collected using a systematic sampling method and include all filed medical claims between 2002 and 2013.

Data from August, 2010 to March, 2012 (pre-policy) and from May, 2012 to December, 2013 (post-policy) were used because the CDCS, defined as the intervention, was implemented on April, 2012. Individuals diagnosed primarily of T2DM (International Classification of Diseases version 10 [ICD-10] E11) were included. The control group included T2DM diagnosed individuals not participating in the CDCS program and the intervention group patients who voluntarily opted to participate in the CDCS program.

The study population comprised of adults aged between 20 and 65 years. Older aged individuals were excluded as they have different levels of disease severity and exhibit unalike treatment patterns. Individuals aged 65 or above are also subject to lower levels of cost sharing for outpatient services that cost up to 15,000 Korean Won [KRW, (1 USD = 1128 KRW)]. Individuals with less than 3 outpatient physician visits were also omitted to ensure consistency in measuring continuity of care. Additionally, Medical Aid beneficiaries were not included as they are subject to different levels of copayment and often have different health care utilization patterns. The final study population consisted of 3388 individuals in the intervention and 14,191 individuals in the control group during the pre-implementation period and 3184 individuals in the intervention and 13,036 individuals in the control group during the post-implementation period.

### Variables

The dependent variables were total health care costs for covered outpatient services and continuity of care. Continuity of care was measured using the usual provider of care (UPC) index, defined as the number of outpatient visits to the most frequently seen physician divided by the total number of outpatient visits [15]. The UPC index concentrates on the number of physicians seen by a patient and the visit ratio of the most frequently seen physician to all visited physicians. All values range between zero and one.

Demographic, socioeconomic, and health related variables were incorporated as covariates. The included variables were age at diagnosis (20 to 29, 30 to 39, 40 to 49, 50 to 59, or 60 to 64), sex (male or female), income (low, middle, or high), region (Seoul, metropolitan, or rural), NHI type (occupational or regional), comorbidities measured using the Charlson Comorbidity Index (0, 1, 2, 3+), disability status (no or yes), medication prescription status (none, insulin only, hypoglycemic agent only, or insulin and hypoglycemic agent), and diabetes related complication status (no or yes). Hypoglycemic agents included sulfonylureas, metformin, alpha glucosidase inhibitors, and thiazolidinedione. Complications refer to retinopathy, nephropathy, neuropathy, peripheral vascular disease, cerebrovascular disease, and cardiovascular disease (CVD) [8].

### Statistical analysis

Quantitative data at baseline and follow up were examined using the chi-square test. The Difference-in-Differences (DID) method was utilized to estimate differences in total health care costs and continuity of care between the intervention and the control group before and after the intervention period. The DID method is a quasi-experimental method that focuses on capturing effects related to a certain event through time between the control and the intervention group [16]. The DID specification of this study were ${y}_{i={\beta}_0+{\beta}_1\bullet {time}_i+\gamma \bullet {group}_i+\delta \bullet {group}_i\bullet {time}_i+{\varepsilon}_i}$. In this regression analysis, *β*_0_ measures the mean outcome of the control group at baseline, *β*_0_+*β*_1_ the mean outcome of the control group at follow up, *γ* the difference between the control and the intervention group at baseline, *β*_0_+ *γ* the mean outcome of the intervention group at baseline, *β*_0_+*β*_1_ + *γ* + *δ* the mean outcome of the intervention group at follow up, *δ* the DID of the intervention, and *ε*_*i*_the random error.

Both adjusted and unadjusted analysis were carried out. All calculated *P* values were two sided, considered significant at < 0.05. Analysis was performed using the SAS software, version 9.4 (SAS Institute, Cary, NC, USA).

## Results

Table 1 shows the general characteristics of the study participants at baseline and follow-up. At the pre-implementation period, a total of 3388 individuals were included in the intervention group and 14,191 individuals in the control group. Similarly, at post-implementation period, 3184 individuals were included in the intervention group and 13,036 individuals in the control group. Characteristics were generally similar between the control and the intervention group, although the control group consisted of a higher percentage of males and a lower percentage of low income individuals. The control group also had a higher proportion of individuals with disability and those diagnosed with diabetes related complications.

**Table 1:** Characteristics of the intervention and the control group before and after policy implementation

	Pre-implementation period				Post-implementation period			
	Control group		Intervention group		Control group		Intervention group	
Total	14,191	(80.7)	3388	(19.3)	13,036	(80.4)	3184	(19.6)
Age								
20–29	144	(1.0)	26	(0.8)	94	(0.7)	15	(0.5)
30–39	928	(6.5)	193	(5.7)	643	(4.9)	112	(3.5)
40–49	3699	(26.1)	883	(26.1)	2856	(21.9)	651	(20.4)
50–59	7183	(50.6)	1780	(52.5)	6238	(47.9)	1580	(49.6)
60–64	2237	(15.8)	506	(14.9)	3205	(24.6)	826	(25.9)
Sex								
Male	8911	(62.8)	2063	(60.9)	8252	(63.3)	1935	(60.8)
Female	5280	(37.2)	1325	(39.1)	4784	(36.7)	1249	(39.2)
Income								
Low	4241	(29.9)	1097	(32.4)	3536	(27.1)	976	(30.7)
Middle	4033	(28.4)	1055	(31.1)	3544	(27.2)	967	(30.4)
High	5219	(36.8)	1230	(36.3)	5208	(40.0)	1239	(38.9)
NHI type								
Occupational	8122	(57.2)	2016	(59.5)	7725	(59.3)	1985	(62.3)
Regional	5371	(37.8)	1366	(40.3)	4563	(35.0)	1197	(37.6)
Region								
Seoul	2670	(18.8)	562	(16.6)	2402	(18.4)	521	(16.4)
Metropolitan	3782	(26.7)	1006	(29.7)	3423	(26.3)	963	(30.2)
Rural	7739	(54.5)	1820	(53.7)	7211	(55.3)	1700	(53.4)
Charlson Comorbidity Index								
0	711.0	(5.0)	149	(4.4)	568.0	(4.4)	115.0	(3.6)
1	6765	(47.7)	1657	(48.9)	6187	(47.5)	1604	(50.4)
2	2573	(18.1)	664	(19.6)	2363	(18.1)	611	(19.2)
3+	4142	(29.2)	918	(27.1)	3918.0	(30.1)	854.0	(26.8)
Disability								
No	12,832	(90.4)	3139	(92.7)	11,684	(89.6)	2940	(92.3)
Yes	1359	(9.6)	249	(7.3)	1352	(10.4)	244	(7.7)
Medication								
None	546	(3.8)	39	(1.2)	476	(3.7)	30	(0.9)
Insulin only	183	(1.3)	45	(1.3)	163	(1.3)	36	(1.1)
Hypoglycemic agent only	12,530	(88.3)	3147	(92.9)	11,544	(88.6)	2964	(93.1)
Insulin & hypoglycemic agent	932	(6.6)	157	(4.6)	853	(6.5)	154	(4.8)
Complication								
No	8258	(58.2)	2050	(60.5)	7626	(58.5)	1966	(61.7)
Yes	5933	(41.8)	1338	(39.5)	5410	(41.5)	1218	(38.3)

*NHI=National Health Insurance

*Hypoglycemic agents include sulfonylureas, metformin, alpha glucosidase inhibitors, and thiazolidinedione*

Table 2 presents the results of the DID model. The effect of the intervention on health care cost was shown through the coefficient of the interaction term post-policy*intervention. Model 1 reveals the effect of the intervention without adjustment for covariates and model 2 with adjustment for covariates. In terms of health care costs, the coefficient of model 1 (β = − 47,523 KRW, *P* < 0.0001) and model 2 (β = − 46,877 KRW, *P* < 0.0001) were statistically significant. This implies a positive relationship between implementation of the CDCS and reduced health care costs for covered outpatient services. Likewise, regarding continuity of care, the coefficient of the interaction term post-policy*intervention was significant in model 1 (β = 0.0537, P < 0.0001) and model 2 (β = 0.0536, P < 0.0001). The results infer that the adoption of the CDCS was associated with improved continuity of care.

**Table 2:** Differential changes in spending and continuity of care between the intervention and the control group

	Total cost						Continuity of Care					
	Model 1			Model 2			Model 1			Model 2		
	β	SE	*p*-value	β	SE	*p*-value	β	SE	*p*-value	β	SE	*p*-value
Pre-policy	Ref			Ref			Ref			Ref		
Post-policy	−15,419	3639	<.0001	−12,789	3665	0.0005	0.0055	0.0016	0.0009	0.0050	0.0017	0.0025
Control group	Ref			Ref			Ref			Ref		
Intervention group	− 9589	3078	0.0018	− 5895	3014	0.0505	0.0093	0.0016	<.0001	0.0093	0.0016	<.0001
Post-policy*Intervention group	−47,523	4700	<.0001	−46,877	4724	<.0001	0.0537	0.0025	<.0001	0.0536	0.0026	<.0001
Age												
20–29				Ref						Ref		
30–39				−63,369	31,611	0.045				0.0070	0.0123	0.5706
40–49				−70,130	31,402	0.0255				0.0118	0.0119	0.3201
50–59				−69,671	31,320	0.0261				0.0130	0.0118	0.2729
60–64				−91,574	31,416	0.0036				0.0139	0.0119	0.2446
Sex												
Male				Ref						Ref		
Female				28,995	5011	<.0001				−0.0037	0.0019	0.0481
Income												
Low				Ref						Ref		
Middle				6009	5507	0.2751				0.0003	0.0023	0.9045
High				7620	4928	0.122				0.0085	0.0021	<.0001
NHI type												
Occupational				Ref						Ref		
Regional				6311	4486	0.1594				−0.0080	0.0018	<.0001
Region												
Seoul				Ref						Ref		
Metropolitan				5500	6125	0.3693				−0.0052	0.0027	0.0525
Rural				6885	5678	0.2253				−0.0049	0.0024	0.0394
Charlson Comorbidity Index												
0				Ref						Ref		
1				−13,878	6915	0.0448				−0.0201	0.0036	<.0001
2				− 4345	8483	0.6086				−0.0196	0.0040	<.0001
3+				10,013	7837	0.2013				−0.0420	0.0039	<.0001
Disability												
No				Ref						Ref		
Yes				35,482	10,247	0.0005				−0.0101	0.0035	0.0035
Medication												
None				Ref						Ref		
Insulin only				−74,185	39,588	0.0609				−0.0375	0.0097	0.0001
Hypoglycemic agent only				− 134,483	21,686	<.0001				−0.0320	0.0040	<.0001
Insulin & hypoglycemic agent				3280	27,210	0.9041				−0.0662	0.0057	<.0001
Complication												
No				Ref						Ref		
Yes				35,446	4322	<.0001				−0.0042	0.0019	0.0304

*NHI=National Health Insurance

*Hypoglycemic agents include sulfonylureas, metformin, alpha glucosidase inhibitors, and thiazolidinedione*

## Discussion

This study investigated the impact of the CDCS on health care costs and continuity of care in Korean adults diagnosed with T2DM. The findings reveal that implementation of the CDCS was associated with reduced expenditures for covered outpatient services and modest improvements in continuity of care. The presented results are in accordance with the expectations of the government, which aimed to incentivize treatment continuity because enhanced coordination has been known to improve quality of care and patient outcomes, which in turn acts to impact the level of health care expenditures [17].

Improvements in continuity of care found in this study are noteworthy because establishing an effective long-term care management plan and improving patient adherence is important in managing chronic diseases. In fact, because chronic diseases cannot be fully cured, patients need to be constantly monitored and well equipped with self-management skills [18]. By improving continuity of care, which familiarizes physicians to patient history, better monitoring of glycemic levels and management of disease related complications can be achieved [19]. At the same time, generating physician-patient trust has also been demonstrated to advance treatment adherence [20]. Correspondingly, previous studies have shown that enhanced treatment continuity can improve patient satisfaction and quality of life, reduce emergency department visits and hospitalizations related to complications, and improve patient outcomes [19, 21, 22]. However, because the primary care system of Korea generally does not function to promote continuity or coordination, many patients are often poorly educated on diabetes-related self-management skills [23]. In fact, the percentage of patients successfully carrying out glucose management have decreased from around 34 to 23% between 2005 and 2014, and almost half of male diabetes patients are reported to smoke [24]. Under such circumstances, implementation of the CDCS may have aided to improve treatment continuity in T2DM patients.

According to the study results, implementation of the CDCS was also related to decreased health care costs for covered outpatient services. Occurrences of T2DM and its related complications have consistently been noted as factors that escalate health care costs [25, 26]. In Korea, macro and micro vascular complications in T2DM patients have specifically been reported to cause higher levels of annual direct medical costs, with expenditures increasing with the number of complications [9]. By incentivizing diabetes patients to utilize primary clinics instead of secondary or tertiary hospitals, the CDCS may have acted to decrease diabetes related health care expenditures by promoting a more effective use of health care resources at the national level. Moreover, the CDCS could have led to reductions in health care costs by inducing treatment continuity considering that better continuity has been previously associated with decreases in health care utilization and expenses [19]. In fact, previous findings have conveyed that poor treatment continuity can escalate risks for diabetic complications and induce heavier health care utilization, which naturally necessitates increases in medical expenditure [19]. Frequent doctor switching may also lead to interruptions in medicine prescription, negatively interfering with patient monitoring and cultivation of self-management skills [3]. As provision of higher quality care is important in managing T2DM patients, this study offers insight by revealing the potential benefits of employing policies that promote a more effective use of health care resources [27].

The findings of this study should be interpreted while accounting for the following limitations. First, not all primary clinics participated in the CDCS program. The program participation rate was around 63% in 2014. Hence, some individuals may have faced difficulties in accessing primary clinics that offer the CDCS program. Second, reductions in total health care costs may have been partially affected by the policy implemented in 2012 that reduced total pharmaceutical costs at an average of around 14%. However, this study did adjust for pharmaceutical prescription status to partially overcome this limitation. Third, health care costs measured in this study only accounted for services reimbursed by the KNHIS. As Korea uses both a positive and a negative list system to determine service reimbursement status, a grey zone of non-covered services that fully depend on patient out of pocket costs exist. As this study only considered costs for covered services due to data limitation, the results cannot be seen as a reflection of total T2DM related costs. Fourth, medical illnesses were identified and categorized solely based on the ICD-10 code. Thus, there may have been some inaccuracies in diagnosis coding. Fifth, pharmaceuticals were classified only based on prescription status, which does not necessarily imply consumption or medical adherence. Last, this study may not have been able to adjust for all potential risk factors, which implies that there may be a possibility of residual confounding. However, despite the limitations stated above, this study is noteworthy as it provides evidence on how implementation of the CDCS affected health care costs and continuity of care in T2DM diagnosed patients.

## Conclusion

This study analyzed the effect of the implementation of the CDCS on health care costs for outpatient services and continuity of care in T2DM patients. The results confirm an association between the adoption of the CDCS and reduced health care costs and improved continuity of care. The discoveries offer insight by signifying the potential benefits of employing effective chronic disease management strategies in reducing health care costs and improving treatment continuity in T2DM patients.

## Abbreviations

CDCS: Chronic Disease Care System
CVD: Cardiovascular disease
DID: Difference in Differences
GP: General practitioner
ICD-10: International Classification of Diseases version 10
KNHIS: Korean National Health Insurance Service
KRW: Korean Won
NHI: National Health Insurance
T2DM: Type 2 Diabetes Mellitus
UPC: Usual provider of care
